# Refractory late-onset cerebrospinal fluid fistula following mammoplasty: case report of a rare complication

**DOI:** 10.31744/einstein_journal/2025RC1610

**Published:** 2025-10-03

**Authors:** Clara Sanches Bueno, Eduardo Carvalhal Ribas, Eduardo Noda Kihara, Andre Felix Gentil, Arthur Werner Poetscher

**Affiliations:** 1 Hospital Israelita Albert Einstein São Paulo SP Brazil Hospital Israelita Albert Einstein, São Paulo, SP, Brazil.; 2 Universidade de São Paulo Hospital das Clínicas Faculdade de Medicina São Paulo SP Brazil Hospital das Clínicas, Faculdade de Medicina, Universidade de São Paulo, São Paulo, SP, Brazil.

**Keywords:** Post-dural puncture headache, Cerebrospinal fluid leak, Blood patch, epidural, Mammoplasty, Intracranial hypotension

## Abstract

Mammoplasty is commonly performed under epidural anesthesia combined with intravenous sedation; however, this carries a risk of dural puncture and post-dural puncture headache. Successful treatment is often achieved with opioids, non-steroidal anti-inflammatory drugs, and caffeine. When conservative treatment fails, an epidural blood patch should be performed; this procedure has a high resolution rate. We report the case of a patient who developed a post-dural puncture headache 25 days after elective breast implant replacement performed under thoracic epidural anesthesia combined with intravenous sedation. The post-dural puncture headache was refractory to conservative treatment and the first epidural blood patch was unsuccessful. This case represents an exceptionally rare presentation of post-dural puncture headache, marked by a delayed onset of nearly four weeks and requiring a second epidural blood patch for symptom resolution.

## INTRODUCTION

Breast augmentation or reconstruction using silicone-based implants is one of the most commonly performed cosmetic procedures worldwide.^(1)^ It is frequently performed under epidural anesthesia combined with intravenous sedation, which is associated with reduced postoperative nausea and vomiting, earlier hospital discharge, and potentially lower costs.^(2)^ However, dural puncture occurs in 0.32-1.23% of cases, with post-dural puncture headache (PDPH) its characteristic symptom.^(3)^ Successful treatment is often achieved with analgesics; refractory cases can be resolved using an epidural blood patch (EBP).^(4)^

Herein, we report a rare case of late-onset PDPH in a patient who had undergone breast implant replacement surgery. The PDPH was refractory to conventional treatment and required repeated EBPs to achieve symptom resolution.

## CASE REPORT

A 40-year-old woman underwent elective breast implant replacement 15 years after her first silicone-based implant surgery. The procedure was performed under thoracic epidural anesthesia at the T4 level, in combination with intravenous sedation, and the patient was discharged with no complications. Specific data regarding the drugs administered during mammoplasty and their respective dosages were not available to our team.

After 25 days of uneventful recovery, the patient began experiencing headaches, predominantly in the nuchal and occipital regions, without irradiation. Musculoskeletal cervicalgia was suspected and treated with a combination of simple painkillers, mild opioids, nonsteroidal anti-inflammatory drugs, and muscle relaxants. A lack of significant improvement led to the patient being referred to our neurosurgical team for further evaluation and management.

**Figure 1:**
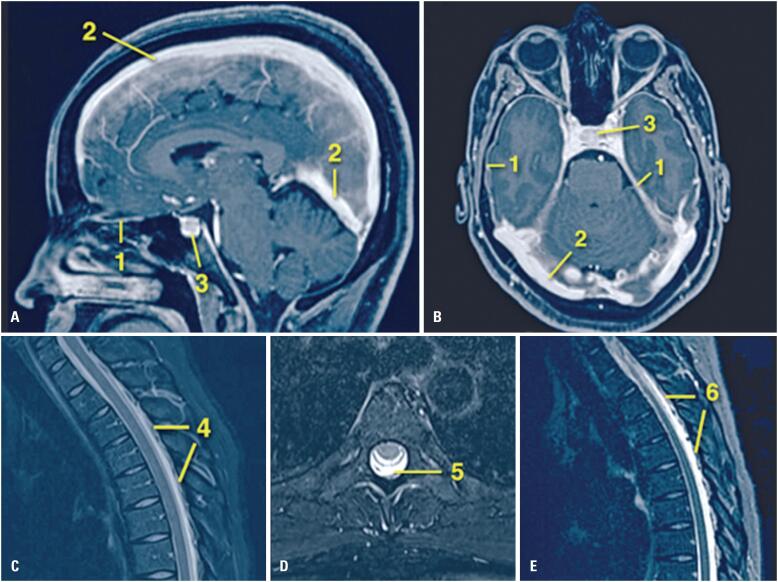
Brain and spinal cord magnetic resonance imaging findings. A) Sagittal and B) axial contrast-enhanced fat-suppressed T1-weighted images reveal findings consistent with CSF hypotension: diffuse and regular thickening and contrast enhancement of the dura mater at the tentorium, supratentorial, and infratentorial compartments (1); enlargement of the dural venous sinuses (2); pituitary engorgement (3); and a slight reduction of some basal cistern spaces. C) A sagittal thoracic spine image in STIR sequence (fat-suppressed T2-weighted equivalent) reveals a collection of laminar posterior epidural fluid spanning T2-T11 (4). D) An axial fat-suppressed T2-weighted sequence shows a maximal collection at T5 (thickness, 3 mm), suggestive of a CSF fistula (5). E) Sagittal post first epidural blood patch follow-up imaging (STIR sequence) demonstrates a persistent but reduced epidural collection (T2-T12), with residual fluid predominance at T4-T5 and T11-T12 (maximum thickness, 2mm) (6)

Further investigation revealed that headaches were triggered in the upright position and improved by sitting or lying down, characteristic of orthostatic headaches caused by cerebrospinal fluid (CSF) hypotension. Magnetic resonance imaging of the brain and spinal cord revealed signs of craniospinal hypotension secondary to an iatrogenic fistula (Figure 1) caused by a dural puncture at the epidural anesthesia site. This was initially treated conservatively with four days of strict bed rest, adequate hydration, and analgesics. However, the patient experienced a recurrence of the orthostatic headache during ambulation following this treatment period.

Computed tomography (CT)-guided EBP was subsequently performed under sedation by an interventional radiology team using a translaminar approach at the T5-T6 level. The procedure was well tolerated until the injection of 6mL autologous blood elicited local pain, at which point it was discontinued with no further complications.

**Figure 2:**
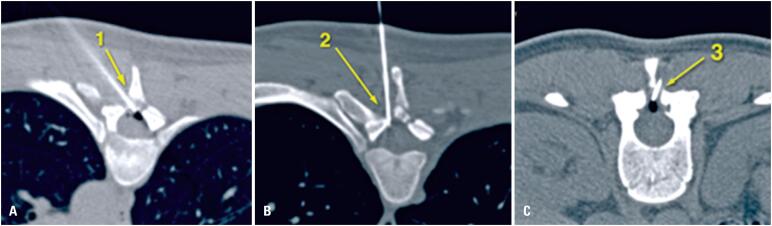
CT-guided interventional epidural blood patches. A) During the first CT-guided epidural blood patch, 6.0mL peripheral blood was injected at the posterior T5-T6 epidural space through a left translaminar approach (1). During the second CT-guided epidural blood patch, B) 15mL peripheral blood was injected at the T3-T4 epidural space (2) and C) 10mL peripheral blood was injected at T12-L1 epidural space (3)

Despite the prescribed bed rest, the patient continued to experience recurrent orthostatic headaches that impaired activities of daily living. A second CT-guided EBP was therefore performed 12 days after the first, this time involving the injection of 15mL autologous blood at the T3-T4 and 10mL at the T12-L1 epidural spaces (Figure 2). The patient remained hospitalized and under strict bed rest for two days, and was then discharged with instructions to maintain bed rest for an additional five days.

Eight months after the second EBP, the patient remained asymptomatic with no reported headaches or neurological symptoms.

This case study was approved by the Research Ethics Committee of *Hospital Israelita Albert Einstein* (CAAE: 45895121.0.0000.0071; # 4.711.940), and written informed consent was obtained from the patient.

## DISCUSSION

Although breast surgery is performed under general anesthesia as standard, adjunctive thoracic epidural anesthesia may be used to provide extended analgesic effects.^(5)^ However, this carries a risk of unintentional dural puncture potentially leading to PDPH. Several risk factors for PDPH have been identified, including female sex, an age of 20-40 years, a low body mass index, previous headaches or PDPH, and operator inexperience.^(6)^ Additionally, spontaneous CSF leakage may result from discogenic pathologies that pierce the dura or connective tissue disorders, although neither etiology was evident in the present case.^(7)^

Post-dural puncture headache occur due to reduced CSF volume and intracranial pressure, with symptoms characteristically worsening in the upright position due to compensatory cerebral vasodilation.^(4)^ In the present case, the patient was female and aged 20-40 years, although no additional risk factors were identified.^(6)^ Classic indicators of dural puncture, such as an exaggerated response to local anesthetics or observable CSF leakage, may be detected during epidural anesthesia; however, they often remain unnoticed, as in the present case.^(8)^

Post-dural puncture headache requires prompt diagnosis and treatment, utilizing opioids, non-steroidal anti-inflammatory drugs, caffeine, and hydration.^(4)^ While symptoms usually manifest within three days following surgery and are often resolved with conservative management within seven days of surgery,^(9)^ studies have documented cases with symptoms presenting as late as postoperative day 14. To the best of our knowledge, no onset later than day 14 has been previously reported in modern literature.^(4)^ Only one study from 1956 reported rare cases of symptom onset months after dural puncture; however, these findings were based on retrospective data with significant limitations and have not been replicated in subsequent studies.^(10)^ The delayed presentation observed in the present case suggests either gradual CSF leakage or an intermittent dural defect, which has been previously described in CSF fistulas.^(7)^

Although PDPH is typically diagnosed using clinical information, magnetic resonance imaging may reveal signs of CSF hypotension and help identify the source of the CSF leak. Additionally, dynamic CT myelography is useful for precisely localizing the sites of rapid CSF leakage; delayed CT myelography or magnetic resonance myelography is often required to detect slow-flow leaks.^(7)^

Epidural blood patch, which was first described in 1960,^(11)^ remains the gold standard treatment for persistent PDPH. It involves injecting 10-30mL autologous blood into the epidural space at or below the leakage site.^(12,13)^ This blood forms a sealing clot that halts further leakage and initiates a local inflammatory response that promotes closure of the dura and restores CSF pressure.^(4,9)^ The success rate of a single EBP is 70-75%, and this rate rises with subsequent patches.^(4,12-14)^ While radiological guidance and larger blood volumes improve efficacy, the injection must be ceased immediately if the patient experiences pain or neurological deficits.^(4)^ Transient back pain occurs in approximately 54% of patients undergoing an EBP, but serious complications are rare.^(12)^

Although the first EBP was performed at the correct site, the procedure was stopped because of local pain, and the suboptimal volume of blood injected prior to this discontinuation likely contributed to treatment failure.^(4)^ While some studies have suggested that volumes of 15-30mL blood may be effective,^(12)^ a consensus regarding the optimal volume is yet to be reached.^(15)^

## CONCLUSION

This case represents a rare presentation of post-dural puncture headache following thoracic epidural anesthesia for mammoplasty, with symptom onset occurring as late as postoperative day 25. This case underscores the critical importance of maintaining clinical suspicion of cerebrospinal fluid leaks when evaluating delayed-onset headaches following epidural anesthesia, to facilitate timely diagnosis and appropriate intervention.
